# The effect of preoperative different dexamethasone regimens on postoperative glycemic control in patients with type 2 diabetes mellitus undergoing total joint arthroplasty: a retrospective cohort study

**DOI:** 10.1186/s13018-023-04485-y

**Published:** 2024-01-03

**Authors:** Ping Mou, Xiao-Dan Zhao, Xin-Yu Cai, Zun-Han Liu, Cheng-Qi He

**Affiliations:** 1 Department of Rehabilitation Medicine, West China Hospital, Sichuan University, #37 Guoxue Road, Chengdu, 610041 People’s Republic of China; 2 Key Laboratory of Rehabilitation Medicine in Sichuan Province, West China Hospital, Sichuan University, #37 Guoxue Road, Chengdu, 610041 People’s Republic of China; 3 Department of Orthopedics, Orthopedic Research Institute, West China Hospital, Sichuan University, #37 Guoxue Road, Chengdu, 610041 People’s Republic of China; 4 Department of Orthopaedic Surgery, Trauma Medical Center, West China Hospital, Sichuan University, Chengdu, 610041 People’s Republic of China; 5 Rehabilitation Medicine Department, Medical Technology Institute, West China Clinical Medical College, Sichuan University, #37 Guoxue Road, Chengdu, 610041 People’s Republic of China; 6 Department of Sports Medicine Center, State Key Laboratory of Trauma, Burn and Combined Injury, Southwest Hospital of the Army Military Medical University, Chongqing, 400038 People’s Republic of China

**Keywords:** Total joint arthroplasty, Dexamethasone, Diabetes mellitus, Glycemic control

## Abstract

**Background:**

Concerns have been raised regarding the impact of preoperative intravenous dexamethasone on postoperative glycemic control in diabetic patients undergoing total joint arthroplasty (TJA). This study aimed to determine relationships between preoperative different dexamethasone regimens and postoperative fasting blood glucose (FBG), as well as to identify risk factors for postoperative FBG ≥ 200 mg/dl in diabetic patients undergoing TJA.

**Methods:**

This retrospective study included 1216 diabetic patients undergoing TJA and categorized into group A (dexamethasone = 0 mg), group B (dexamethasone = 5 mg), and group C (dexamethasone = 10 mg). All dexamethasone was administered before skin incision. FBG levels were monitored until postoperative day (POD) 3. Analyses were conducted for periprosthetic joint infection (PJI) and wound complications during 90 days postoperatively. And the risk factors for postoperative FBG ≥ 200 mg/dl were identified.

**Results:**

Preoperative dexamethasone administration resulted in a transiently higher FBG on POD 0 and POD 1 (all *P* < 0.001). However, no differences were observed on POD 2 (*P* = 0.583) and POD 3 (*P* = 0.131) among three groups. While preoperative dexamethasone led to an increase in postoperative mean FBG and postoperative maximum FBG (all *P* < 0.001), no differences were found in wound complications (*P* = 0.548) and PJI (*P* = 1.000). Increased HbA1c and preoperative high FBG, but not preoperative dexamethasone, were identified as risk factors for postoperative FBG ≥ 200 mg/dl. Preoperative HbA1c level of ≥ 7.15% was associated with an elevated risk of postoperative FBG ≥ 200 mg/dl.

**Conclusions:**

Although preoperative intravenous administration of 5 mg or 10 mg dexamethasone in diabetic patients showed transient effects on postoperative FBG after TJA, no differences were found in the rates of PJI and wound complications during 90 days postoperatively. Notably, patients with a preoperative HbA1c level of ≥ 7.15% and elevated preoperative FBG may encountered postoperative FBG ≥ 200 mg/dl.

## Background

Type 2 diabetes mellitus (DM) is an age-related disease and accounts for nearly 90% of the approximately 537 million global diabetic cases [1]. The rising worldwide incidence of DM is obvious, which is estimated to rise to 783 million by 2045 [2]. Total joint arthroplasty (TJA) including total hip arthroplasty (THA) and total knee arthroplasty (TKA) is conventionally performed in elderly patients diagnosed with end-stage arthropathies to alleviate pain and restore joint function. The prevalence of DM in TJA patients is estimated to surpasses 15% for THA and 21% for TKA [3]. And the prevalence of DM and perioperative hyperglycemia correlate with increased postoperative complications, including prosthetic joint infection (PJI) and wound complications [4, 5]. Studies have identified postoperative blood glucose levels ≥ 200 mg/dl (11.1 mmol/L) as a risk factor for surgical site infections, even in patients with preoperatively well-controlled blood glucose levels [6, 7]. Hence, postoperative blood glucose levels should be well-managed to avoid adverse events in diabetic patients undergoing TJA.

However, TJA intervention and anesthesia may induce metabolic disorders, resulting in poorly controlled postoperative blood glucose, particularly in diabetic patients [8, 9]. Additionally, operation-related soft tissue injury can trigger systemic inflammatory response and insulin resistance to exacerbate challenges in glycemic control [10]. Dexamethasone, a potent and long-acting glucocorticoid, is routinely administered intravenously preoperatively in TJA patients to attenuate perioperative acute phase response and alleviate postoperative pain, nausea, and vomiting [11–13], which were beneficial for glycemic control. But, dexamethasone inherently induces hyperglycemia [4], inhibits the inflammatory phase of wound healing [14], and suppresses the immune system [15], which potentially increase the risk of postoperative hyperglycemia and surgical site infections. The dual nature of dexamethasone, with both positive and negative effects, prompts an exploration of its comprehensive impact on glycemic control in type 2 DM patients undergoing TJA. Several studies have discussed the relationships of dexamethasone to blood glucose in diabetic patients undergoing TJA and they preliminarily concluded that an initial increase in blood glucose was observed after dexamethasone administration, yet there was no increase in the rate of infection [9, 16–19]. However, the current studies were limited by different dexamethasone administration ways [16], different dexamethasone doses [18], small sample size [9, 16, 17], non-diabetic patients included [16, 18], and oversight of food effects [19]. Consequently, further study was needed to identify the effect of dexamethasone on blood glucose levels in diabetic patients undergoing TJA.

This study aimed to evaluate the influence of preoperative intravenous administration of different doses of dexamethasone on postoperative blood glucose levels in diabetic patients undergoing TJA. Furthermore, we determined the risk factors associated with elevated blood glucose levels (≥ 11.1 mmol/L) during the admission period. We hypothesized that compared to the non-dexamethasone group, preoperative intravenous dexamethasone yielded transient effects on increasing the blood glucose levels and dexamethasone administration was not associated with blood glucose levels ≥ 11.1 mmol/L.

## Methods

### Study design and patient enrollment

This retrospective study was conducted in West China hospital, Sichuan University. The study was approved by the Clinical Trials and Biomedical Ethics Committee of hospital. All procedures followed were performed in accordance with the Declaration of Helsinki in 1975 and with the ethical standards of the responsible committee on human experimentation (institutional or regional).

**Fig.1:**
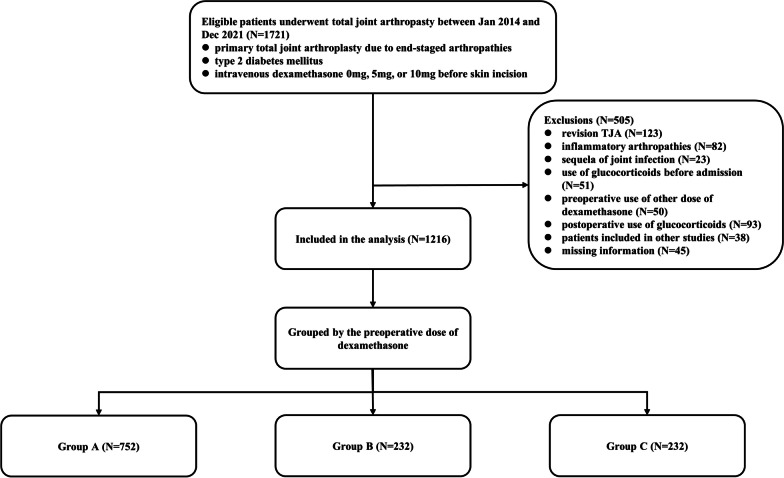
Flow diagram of the study design

Patients were included retrospectively if they were diagnosed with type 2 DM and performed primary TJA due to end-staged arthropathies in our joint reconstruction center between January 2014 and December 2021. Among the eligible patients, patients were excluded if they had: revision TJA, diagnose of inflammatory arthropathies or sequela of joint infection, use of glucocorticoids before admission or in the postoperative periods, preoperative use of other dose of dexamethasone, participation in other studies, and missing information. Finally, 1216 patients were included. Patients were stratified into three cohorts including group A, those preoperative intravenous administration of 0 mg dexamethasone (N = 752), group B, those preoperative intravenous administration of 5 mg dexamethasone (N = 232), and group C, those preoperative intravenous administration of 10 mg dexamethasone (N = 232) (Fig. 1).

### Perioperative management

All patients were performed TJA under general anesthesia. In group A, dexamethasone was not used during the perioperative period. And in group B and group C, dexamethasone was only administered intravenously before skin incision was made. All patients were performed standardized perioperative care. Multimodal analgesia protocols were managed to control pain. Preoperatively, oral celecoxib was scheduled to conduct preemptive analgesia after admission. Intraoperatively, local infiltration anesthesia with bupivacaine was injected into the capsular and soft tissues during the procedure and before closure. Postoperatively, a cold pack was used to decrease pain on the surgical site for approximately 12 h. Oral celecoxib and oxycodone hydrochloride tablet were served as postoperative analgesia. Ondansetron was used for postoperative nausea and vomiting prophylaxis. Physical therapy and functional exercise were initiated immediately when patients recovered from anesthesia. For the prophylaxis of deep vein thrombosis, 2000 IU enoxaparin was routinely scheduled subcutaneously 6 h postoperatively, and 4000 IU enoxaparin was given at 24-h intervals until discharge. After discharge, the patients undergoing THA and TKA were routinely received oral rivaroxaban for 10 days [20, 21].

The perioperative blood glucose goal was from 6.7 to 10 mmol/L. Glycemic control in diabetic patients was achieved by a standardized protocol including diabetic diet, patient’s home diabetic medications, and temporary short-acting insulin in hospital administered on a sliding scale with intervention beginning at glucose level more than 11.1 mmol/L.

### Data collection and outcome measures

Data were collected from our institution’s electronic medical record. Baseline demographics and clinical characteristics were collected. Preoperative glycated hemoglobin (HbA1c) was the nearest HbA1c to the date of TJA (within 3 months). Preoperative fasting blood glucose (FBG) was measured in the morning of the day before TJA. FBG in postoperative day (POD) 0 returning to ward immediately was measured when patients returning ward after surgery. And FBG in POD 0, POD 1, POD 2, and POD 3 was measured before meals in the morning. FBG during hospital stay was the average of FBGs including preoperatively, POD 0, POD 0 returning to ward immediately, POD 1, POD 2, and POD 3. The occurrences of PJI and wound complication in 90 days postoperatively were recorded. PJI was diagnosed according to the criteria published and modified by the Musculoskeletal Infection Society [22]. Additionally, wound complications was diagnosed in accordance with Centers for Disease Control definitions of nosocomial surgical site infections in 1992 [23].

### Statistical analysis

Continuous data were presented as the mean and standard deviation (SD) and categorical data were presented as numbers and percentages. One-way analysis of variance with post-hoc LSD test was used for continuous variables. Chi-square or Fisher’s tests was used for categorical variables. Univariate analysis was used to compare baseline demographic and clinical characteristic. Studies also have identified postoperative FBG levels ≥ 200 mg/dl (11.1 mmol/L) as a risk factor for surgical site infections, even in patients with preoperatively well-controlled blood glucose levels [6, 7]. So, any variables with *p*-value < 0.10 related to postoperative FBG levels ≥ 11.1 mmol/L were identified as potential predictors and included in a multivariate logistic regression analysis to determine independent risk factors. The dexamethasone was included in the multivariate logic regression analysis regardless of the p-value of the univariate analysis. The results were reported as odds ratios (OR) with 95% confidence intervals (CI). Significance was set at *p* < 0.05.

The receiver operating characteristic curves were generated to describe the relationships between the true-positive rate (sensitivity) and false-positive rate (1-specificity), as well as to calculate area under the curve (AUC). The Youden index was used to determine the optimal predictive cutoffs for the tested markers to determine the relationships between the preoperative HbA1c level threshold and postoperative blood glucose ≥ 11.1 mmol/L. All statistical analyses were performed using IBM SPSS Statistics version 24.

## Results

### Patient flow and baseline demographics

A total of 1721 diabetic patients undergoing TJA were assessed for eligibility. Finally, 1216 patients were included and the data were collected and analyzed for these patients. 752 patients were grouped into group A, 232 patients were grouped into group B, and 232 patients were grouped into group C. There was no difference in baseline demographic and clinical characteristics (Table 1). Specially, no differences were found on preoperative HbA1c (*P* = 0.145) and FBG (*P* = 0.537).

**Table 1:** Baseline demographics and clinical characteristics

Characteristics	Group A (dexamethasone = 0 mg)	Group B (dexamethasone = 5 mg)	Group C (dexamethasone = 10 mg)	*P* value
Demographic characteristics				
Age (years old)	66.8 ± 8.5	68.0 ± 7.8	67.3 ± 9.3	0.189
Gender (M/F)	176/576	46/186	51/181	0.512
Height (cm)	158.7 ± 8.0	156.9 ± 7.7	157.7 ± 7.9	0.006*
Weight (kg)	65.3 ± 10.1	63.6 ± 9.8	63.4 ± 10.9	0.012*
BMI (kg/m^2^)	25.9 ± 3.3	25.8 ± 3.2	25.5 ± 3.6	0.241
Smoker (Yes)	115 (15.3%)	24 (10.3%)	27 (11.6%)	0.097
Alcohol user (Yes)	134 (17.8%)	33 (14.2%)	29 (12.5%)	0.107
Procedure (Knee/Hip)	474/278	164/68	150/82	0.102
Operated sides (L/R)	413/339	120/112	118/114	0.460
ASA classification				
I	0 (0%)	0 (0%)	0 (0%)	0.638
II	550 (73.1%)	165 (71.1%)	163 (70.3%)	
III	202 (26.9%)	67 (28.9%)	69 (29.7%)	
IV	0 (0%)	0 (0%)	0 (0%)	
Primary diagnosis				0.140
OA	514 (68.4%)	178 (76.7%)	155 (66.8%)	
DDH	131 (17.4%)	27 (11.6%)	43 (18.5%)	
Dated fracture	8 (1.1%)	0 (0%)	5 (2.2%)	
ONFH	86 (11.4%)	24 (10.3%)	24 (10.3%)	
Traumatic OA	13 (1.7%)	3 (1.3%)	5 (2.2%)	
Laboratory examinations				
Preoperative HbA1c (%)	7.0 ± 1.1	6.9 ± 1.0	7.1 ± 1.1	0.145
Preoperative FBG (mmol/L)	7.2 ± 2.2	7.1 ± 1.9	7.3 ± 2.1	0.537
Preoperative ESR (mm/h)	26.5 ± 14.4	28.0 ± 13.2	27.0 ± 12.9	0.354
Preoperative CRP (mg/L)	3.7 ± 2.4	3.6 ± 2.6	3.5 ± 2.2	0.459
Preoperative hemoglobin (g/L)	128.1 ± 12.5	126.9 ± 14.6	125.9 ± 14.6	0.079
Preoperative albumin (g/L)	43.2 ± 3.5	43.5 ± 3.7	43.8 ± 3.7	0.078
Hospital expense (RMB)	57,521.5 ± 13,600.4	57,988.8 ± 19,440.5	59,659.7 ± 14,971.7	0.171

M = male; F = female; BMI = body mass index; L = left; R = right; ASA = American society of aneshesiologists; OA = osteoarthritis; DDH = developmental dysplasia of the hip; ONFH = osteonecrosis of the femoral head; HbA1c = glycated hemoglobin; FBG = fasting blood glucose; ESR = erythrocyte sedimentation rate; CRP = C-reaction protein; RMB = ren min bi; 11.1 mmol/L = 200 mg/dl. * meant *p* < 0.05 and the values were marked in bold

### Postoperative blood glucose levels

**Fig. 2:**
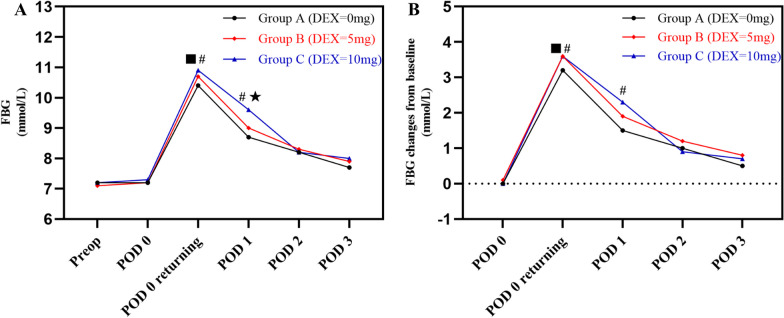
**A** The level of FBG and FBG changes preoperatively and postoperatively. FBG = fasting blood glucose; POD = postoperative day. **B** The level of FBG change from baseline postoperatively. FBG = fasting blood glucose; POD = postoperative day. ■ meant there was statistical difference between group A and B. # meant there was statistical difference between group A and C. ★ meant there was statistical difference between group B and C

Compared to group A, preoperative intravenous use of dexamethasone transiently increased blood glucose levels in diabetic patients after TJA on POD 0 and 1. When the patients returned to ward immediately, the FBG for group B and group C were higher than that for group A (*P*1 = 0.013 and *P*2 < 0.001). However, no difference was found on group B and C (*P*3 = 0.260). On POD 1, the FBG for group C was higher than that for group A and group B (*P*2 < 0.001 and *P*3 = 0.006). And there was no difference on group A and B (P1 = 0.189). There was no significant difference in the three cohorts On POD 2 (*P* = 0.583) and POD 3 (*P* = 0.131). The FBG for group C was higher than that for group A (P2 = 0.005) during hospital stay, while there were no differences between group B and group A (*P*1 = 0.262) or group C (*P*3 = 0.173) (Table 2 and Fig. 2A).

**Table 2:** Postoperative blood glucose levels after TJA

Characteristics	Group A	Group B	Group C	*P* value	*P*1	*P*2	*P*3
FBG (mmol/L)							
POD 0	7.2 ± 1.6	7.2 ± 1.6	7.3 ± 1.5	0.574	0.879	0.294	0.468
POD 0 returning to ward immediately	10.4 ± 1.8	10.7 ± 1.7	10.9 ± 2.1	< 0.001*	0.013*	< 0.001*	0.260
POD 1	8.7 ± 2.2	9.0 ± 2.5	9.6 ± 2.6	< 0.001*	0.189	< 0.001*	0.006*
POD 2	8.2 ± 1.6	8.3 ± 1.9	8.2 ± 1.9	0.583	0.303	0.908	0.459
POD 3	7.7 ± 1.5	7.9 ± 1.8	8.0 ± 1.7	0.131	0.328	0.052	0.435
During hospital stay	8.2 ± 1.4	8.4 ± 1.5	8.5 ± 1.5	0.018*	0.262	0.005*	0.173
FBG changes from preoperative baseline (mmol/L)							
POD 0	0 ± 1.2	0.1 ± 0.9	0 ± 1.3	0.380	0.170	0.914	0.306
POD 0 returning to ward immediately	3.2 ± 1.8	3.6 ± 1.7	3.6 ± 2.2	< 0.001*	0.001*	0.003*	0.887
POD 1	1.5 ± 2.3	1.9 ± 2.4	2.3 ± 2.6	< 0.001*	0.060	< 0.001*	0.085
POD 2	1.0 ± 1.9	1.2 ± 1.9	0.9 ± 2.1	0.144	0.103	0.493	0.061
POD 3	0.5 ± 1.9	0.8 ± 2.0	0.7 ± 2.1	0.297	0.137	0.424	0.578
During hospital stay	1.2 ± 1.5	1.5 ± 1.5	1.5 ± 1.7	0.019*	0.018*	0.042*	0.787
Postoperative mean FBG (mmol/L)	8.8 ± 1.5	9.0 ± 1.7	9.2 ± 1.7	< 0.001*	0.075	0.001*	0.169
Postoperative maximum FBG (mmol/L)	10.7 ± 1.9	11.0 ± 2.0	11.4 ± 2.3	< 0.001*	0.020*	< 0.001*	0.055
Postoperative maximum FBG ≥ 11.1 mmol/L (%)	269 (35.8%)	91 (39.2%)	104 (44.8%)	0.043*	0.381	0.016*	0.259

TJA = total joint arthroplasty; DEX = dexamethasone; FBG = fasting blood glucose; POD = postoperative day. 11.1 mmol/L = 200 mg/dl. *P* = *p*-value of Group A versus B versus C; *P*1 = *p*-value of Group A versus B; *P*2 = *p*-value of Group A vs C; P3 = *p*-value of Group B versus C

*Meant *p* < 0.05 and the values were marked in bold

The FBG changes from preoperative baseline showed that the patients for group B and group C encountered higher FBG changes compared to group A when the patients returned to ward immediately (*P*1 = 0.001 and *P*2 = 0.003), While there was no difference between group B and group C (*P*3 = 0.887). On POD 1, compared to group A, The FBG changes from preoperative baseline for group C were higher (*P*2 < 0.001), While no differences were found on group A and group B (*P*1 = 0.060) or group B and group C (*P*3 = 0.085). There were no differences on FBG changes from preoperative baseline in the three cohorts on POD 2 (*P* = 0.144) and POD 3 (*P* = 0.297). The FBG changes from preoperative baseline for group A were lower than that for group B (*P*1 = 0.018) and group C (*P*2 = 0.042) during hospital stay, while there was no difference between group B and group C (*P*3 = 0.787) (Table 2 and Fig. 2B).

The postoperative mean FBG was (8.8 ± 1.5) mmol/L for group A, (9.0 ± 1.7) mmol/L for group B, and (9.2 ± 1.7) mmol/L for group C. The postoperative mean FBG for group C was higher than that for group A (*P*2 = 0.001), while no differences were found for group A and group B (*P*1 = 0.075) or group B and group C (*P*3 = 0.169). The postoperative maximum FBG was (10.7 ± 1.9) mmol/L for group A, (11.0 ± 2.0) mmol/L for group B, and (11.4 ± 2.3) mmol/L for group C. The postoperative maximum FBG for group A was lower than that for group B (*P*1 = 0.020) and group C (*P*2 < 0.001). Additionally, more patients in group C encountered postoperative maximum FBG ≥ 11.1 mmol/L than that in group A (*P*2 = 0.016), while no differences were found in group A and group B (*P*1 = 0.381) or group B and group C (*P*3 = 0.259) (Table 2).

For diabetic patient undergoing THA, there were differences among three groups on FBG levels when the patients returned to ward immediately (*P* = 0.005) and on POD 1 (*P* < 0.001). Furthermore, no differences were found among three groups on POD2 (*P* = 0.615), POD3 (*P* = 0.172), and during hospital stay (*P* = 0.085). The postoperative mean FBG was (8.6 ± 1.4) mmol/L for group A, (8.5 ± 1.4) mmol/L for group B, and (9.0 ± 1.8) mmol/L for group C and no differences were found among three groups (*P* = 0.108). Although there were differences on postoperative maximum FBG (*P* = 0.002), no differences were found on the rate of postoperative maximum FBG ≥ 11.1 mmol/L (*P* = 0.141) (Table 3).

**Table 3:** Postoperative blood glucose levels after THA and TKA

	THA				TKA			
	Group A	Group B	Group C	*P* value	Group A	Group B	Group C	*P* value
FBG (mmol/L)								
POD 0	7.3 ± 1.6	6.8 ± 1.4	7.3 ± 1.6	0.101	7.2 ± 1.6	7.4 ± 1.6	7.3 ± 1.4	0.270
POD 0 returning to ward immediately	10.2 ± 1.7	10.3 ± 1.7	11.0 ± 2.3	0.005*	10.5 ± 1.8	10.9 ± 1.7	10.9 ± 2.0	0.006*
POD 1	8.4 ± 2.0	8.4 ± 2.4	9.2 ± 2.6	< 0.011*	8.9 ± 2.3	9.2 ± 2.6	9.8 ± 2.6	0.001*
POD 2	8.1 ± 1.6	7.9 ± 1.6	7.9 ± 2.0	0.615	8.2 ± 1.6	8.5 ± 2.0	8.3 ± 1.8	0.300
POD 3	7.8 ± 1.6	7.4 ± 1.4	7.7 ± 1.6	0.172	7.7 ± 1.5	8.1 ± 1.8	8.1 ± 1.8	0.012*
During hospital stay	8.2 ± 1.4	7.9 ± 1.3	8.4 ± 1.6	0.085	8.3 ± 1.4	8.5 ± 1.6	8.6 ± 1.5	0.013*
Postoperative mean FBG (mmol/L)	8.6 ± 1.4	8.5 ± 1.4	9.0 ± 1.8	0.108	8.8 ± 1.5	9.2 ± 1.7	9.3 ± 1.6	0.004*
Postoperative maximum FBG (mmol/L)	10.4 ± 1.7	10.6 ± 2.1	11.3 ± 2.5	0.002*	10.8 ± 2.0	11.2 ± 2.0	11.4 ± 2.2	0.003*
Postoperative maximum FBG ≥ 11.1 mmol/L (%)	91 (32.7%)	18 (26.5%)	34 (41.5%)	0.141	178 (37.6%)	73 (44.5%)	70 (46.7%)	0.076

THA = total hip arthroplasty; TKA = total knee arthroplasty; DEX = dexamethasone; FBG = fasting blood glucose; POD = postoperative day. 11.1 mmol/L = 200 mg/dl

*Meant *p* < 0.05 and the values were marked in bold

For diabetic patient undergoing TKA, there were differences among three groups on FBG levels when the patients returned to ward immediately (*P* = 0.006), on POD 1 (*P* = 0.001), on POD 3 (*P* = 0.012), and during hospital stay (0.013). Furthermore, no differences were found among three groups on POD2 (*P* = 0.300). The postoperative mean FBG was (8.8 ± 1.5) mmol/L for group A, (9.2 ± 1.7) mmol/L for group B, and (9.3 ± 1.6) mmol/L for group C and differences were found among three groups (*P* = 0.004). Although there were differences on postoperative maximum FBG (*P* = 0.003), no differences were found on the rate of postoperative maximum FBG ≥ 11.1 mmol/L (*P* = 0.076) (Table 3).

### Risk factors for postoperative FBG level ≥ 11.1 mmol/L and AUC of glucose

**Fig.3:**
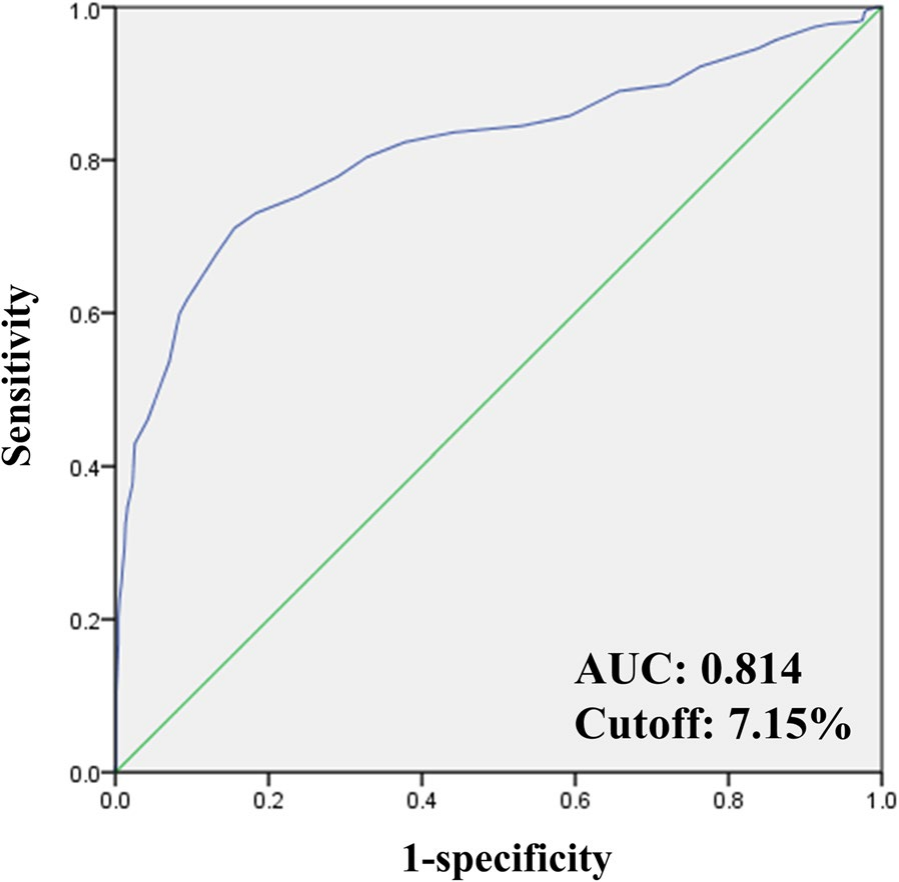
Receiver operating characteristic curve to determine the preoperative HbA1c level threshold, which increases the risk for postoperative blood glucose ≥ 11.1 mmol/L after TJA. AUC = area under the curve; TJA = total joint arthroplasty

After univariate analysis, dexamethasone, gender, smoking status, the procedures, preoperative HbA1c, and preoperative FBG were included in the final model for the multivariable analysis. The results showed that the elevated preoperative HbA1c (OR: 2.843; 95% CI: 2.232–3.621; *P* < 0.001) and the increasing preoperative FBG (OR: 1.412; 95% CI: 1.250–1.596; *P* < 0.001) were the risk factors for postoperative FBG levels ≥ 11.1 mmol/L. However, both preoperative administration of 5 mg and 10 mg dexamethasone did not increase the risk for postoperative FBG levels ≥ 11.1 mmol/L (Table 4). The preoperative HbA1c level threshold, which increased the risk for postoperative FBG levels ≥ 11.1 mmol/L, was 7.15% (Fig. 3). The area under the curve was 0.814 (95% CI: 0.787–0.842; *P* < 0.001).

**Table 4:** Logistic regression of risk factors for postoperative FBG level ≥ 200 mg/dl during the postoperative period of TJA

Variables	Univariate analysis		Multivariate analysis	
	OR (95%CI)	*P* value	OR (95%CI)	*P* value
Preoperative dexamethasone				
0 mg	Reference	–	Reference	–
5 mg	1.159(0.856–1.569)	0.340	1.297(0.892–1.884)	0.173
10 mg	1.459(1.082–1.967)	0.013*	1.380(0.958–1.986)	0.084
Age (years old)	1.005(0.991–1.019)	0.471		
Female	1.424(1.070–1.895)	0.015*	1.412(0.905–2.202)	0.129
BMI (kg/m^2^)	1.008(0.973–1.044)	0.655		
Smoker	0.729(0.514–1.034)	0.076*	0.775(0.454–1.324)	0.351
Alcohol user	0.793(0.575–1.095)	0.159		
Procedures				
TKA	Reference	–	Reference	–
THA	0.730(0.571–0.934)	0.012*	0.749(0.544–1.032)	0.077
ASA classificaiton				
II	Reference	–		
III	0.917(0.707–1.189)	0.512		
Primary diagnosis				
OA	Reference	–		
DDH	0.847 (0.615–1.167)	0.310		
Dated fracture	2.482 (0.805–7.651)	0.114		
ONFH	0.733 (0.497–1.080)	0.117		
Traumatic OA	1.410 (0.592–3.357)	0.437		
Laboratory examinations				
Preoperative HbA1c (%)	4.639 (3.843–5.600)	< 0.001*	2.843 (2.232–3.621)	< 0.001**
Preoperative FBG (mmol/L)	2.028 (1.844–2.231)	< 0.001*	1.412 (1.250–1.596)	< 0.001**
ESR (mm/h)	1.007 (0.999–1.016)	0.085*	1.001 (0.991–1.012)	0.826
CRP (mg/L)	1.030 (0.981–1.080)	0.232		
Hemoglobin (g/L)	1.006 (0.997–1.015)	0.166		
Albumin (g/L)	1.005 (0.973–1.038)	0.761		

TJA = total joint arthroplasty; OR = odds ratio; CI = confidence interval; BMI = body mass index; TKA = total knee arthroplasty; THA = total hip arthroplasty; ASA = American Society of Anesthesiologists classifications; OA = osteoarthritis; DDH = developmental dysplasia of the hip; ONFH = osteonecrosis of the femoral head; ESR = erythrocyte sedimentation rate; CRP = C-reaction protein; 11.1 mmol/L = 200 mg/dl

*Meant *p* < 0.10 for univariate analysis, which were included in multivariate analysis

**Meant *p* < 0.05 and the values were marked in bold

### Clinical outcomes

5 (0.7%) patients in group A encountered wound complications and 2 (0.3%) patients in group A encountered PJI and readmitted to hospital within 90 days postoperatively. And all of the complications were treated successfully. No patients in group B and group C encountered wound complications or PJI within 90 days postoperatively. Moreover, there were no differences for the prevalence of wound complications (*P* = 0.548) and PJI (*P* = 1.000).

## Discussion

The elevated blood glucose levels were associated with complications after TJA, particularly in diabetic patients [9, 18, 19]. Consequently, the orthopedic surgeons have exhibited reluctance in intravenously administering dexamethasone to patients during the perioperative period. This study aimed to evaluate the correlations between preoperative different dexamethasone administration and postoperative blood glucose levels in diabetic patients undergoing TJA. Additionally, the study also identified the risk factors for postoperative hyperglycemia. The principal finding was that preoperative intravenous administration of 5 mg or 10 mg dexamethasone in diabetic patients undergoing TJA would lead to a transient increase in FBG. However, this increase did not yield clinically significant difference and promptly normalized during the subsequent assessments. Furthermore, no differences were found on the rates of PJI and wound complications within 90 days postoperatively. Our study also found that increased HbA1c and preoperative high FBG, but not preoperative dexamethasone administration, worked as the predictive indicators for postoperative hyperglycemia.

Anesthesia and surgery can lead to stress response and contribute to marked neurophysiological changes characterized by the releases of many hormones, such as cortisol, glucagon, and growth hormone, which can elevate the glucose levels and augment insulin resistance [24]. Additionally, diabetic patients often presented with disruptions in glucose metabolism. So, the use of dexamethasone in diabetic patients undergoing TJA may precipitate postoperative hyperglycemia and substantial changes in blood glucose levels. Postoperative hyperglycemia was associated with poor postoperative outcomes in orthopedic surgery including infection, mortality, and poor rehabilitation [24, 25]. Currently, several studies have focused on the effect of dexamethasone on glycemic control in diabetic patients after TJA. Nurok et al. [16] found that there was no evidence of an association between dexamethasone administration and the odds of postoperative glucose levels ≥ 200 mg/dl or higher maximum glucose levels. Park et al. [9] further confirmed that patients with a preoperative HbA1c level of ≥ 7.05% may necessitate alterations in diabetic medication after TKA, regardless of intravenous dexamethasone administration. And Godshaw [18] et al. demonstrated perioperative intravenous dexamethasone did not increase the rate of PJI. Moreover, Allen et al. [17] and Arraut et al. [19] concluded that dexamethasone was associated with transient increase in blood glucose among diabetic patients after TJA, yet this unfavorable effect may not outweigh the known clinical benefits of perioperative glucocorticoids. In our study, we also found preoperative administration of 5 mg or 10 mg dexamethasone to diabetic patients was linked to an initial increase in blood glucose after TJA. Importantly, this increase does not increase the rates of PJI and wound complications within the 90-day postoperative period. Discrepancies with Nurok et al. [16], we found that dexamethasone would lead to higher maximum glucose levels in diabetic patients after TJA. Because Nurok et al. [16] included many non-diabetic patients in dexamethasone group and the diabetic individuals were more likely to experience disturbance of glucose metabolism after TJA. Also, Nurok et al. [16] also acknowledged that their findings may not be generalizable to patients having different baseline characteristics or procedures. So, these may be the reasons why our results were different.

The degree of metabolic disorders correlated with the dose of glucocorticoid [26]. So, whether intravenous 10 mg dexamethasone can result in greater metabolic disturbance than 5 mg dexamethasone in diabetic patients after TJA? Several studies have reported the effect of dexamethasone on FBG levels in diabetic patients after TJA. Allen et al. [17] found that there was no statistically significant association between preoperative 8 mg dexamethasone administration and maximum blood glucose levels within 24 h after surgery. Additionally, Park et al. [9] reported that 10 mg dexamethasone transiently increased blood glucose levels in diabetic patients on POD 0 and POD 1, but had no effect beyond POD 1. Godshaw et al. [18] demonstrated that although diabetic patients experienced significantly higher glucose levels than non-diabetic ones after administration of 12 mg dexamethasone, this was unaffected by the administration of dexamethasone. So, dexamethasone can elevate blood glucose levels in diabetic patients after TJA. Arraut et al. [19] further compared the effects of intravenous 10 mg dexamethasone with 20 mg dexamethasone on glucose levels in diabetic patients and they found that patients after administration of 20 mg dexamethasone trended towards slightly higher mean postoperative blood glucose levels at 24–36 h postoperatively. But, there was no significant difference on maximum blood glucose levels within 72 h. And there were no differences on postoperative glucose levels over 200 mg/dL. So, higher dose of dexamethasone would be more likely to elevate postoperative blood glucose. But, this transient tendency did not lead to clinical difference. Similarly, our study also found that preoperative dexamethasone can elevate FBG levels within POD 1 and there were no differences on postoperative mean FBG levels.

Intravenous dexamethasone can reduce postoperative pain, opioid consumption, nausea/vomiting for patients undergoing TJA [27]. Since glucocorticoid may potentially disturb the blood glucose, the studies evaluating the effect of dexamethasone in TJA excluded the diabetic patients due to the concern of possibly excessive blood glucose fluctuations. Evidence-based guidelines for dexamethasone use in TJA advocated for further research exploring the safety aspects of its administration in patients with DM [27]. Multiple studies have showed that blood glucose levels ≥ 200 mg/dl (11.1 mmol/L) can increase the risk of postoperative infetion and wound complications [9, 17–19]. Therefore, it was important to distinguish the risk factors for blood glucose levels ≥ 11.1 mmol/L. Allen et al. [17] identified elevated HbA1c and advancing age as risk factors for blood glucose levels ≥ 11.1 mmol/L. Similarly, Park et al. [9] demonstrated that high preoperative HbA1c levels increased and high body mass index decreased the postoperative blood glucose levels. Additionally, Nurok et al. [16] reported no evidence of an association between preoperative pain or postoperative pain and the odds of postoperative blood glucose levels ≥ 11.1 mmol/L. All these studies concluded that preoperative dexamethasone did not increase the occurrence of postoperative blood glucose levels ≥ 11.1 mmol/L. Consistent with these findings, our study also revealed that high preoperative HbA1c increased the risk of postoperative hyperglycemia, while there was no association between preoperative administration of 5 mg or 10 mg dexamethasone and blood glucose levels ≥ 11.1 mmol/L. Notably, if the preoperative HbA1c level was more than 7.15%, the patients may be susceptible to blood glucose levels ≥ 11.1 mmol/L. Moreover, we firstly reported that high preoperative FBG contributed to a high risk of blood glucose levels ≥ 11.1 mmol/L. High preoperative FBG can reflect the poor control of recent blood glucose levels. And postoperative blood glucose may be more likely influenced by the surgery and so on. This may be the reason why high preoperative FBG was associated with postoperative hyperglycemia.

PJI, a devastating complication associated with increased morbidity and mortality, necessitates subsequent multiple operations and requires substantial health-care funds [28]. And wound complications are the predominant causes of readmission in Asian population [29]. Wound problems can result in disastrous consequence and how to achieve effective wound healing in TJA is essential to improve satisfaction levels. Therefore, it’s important to avoid PJI and wound complications after TJA. But for diabetic patients scheduled to TJA, DM has been identified as an independent risk factor for PJI with study showing 2–3 times the risk [18]. Moreover, diabetic individuals were more likely to experience wound complication [30]. Given these considerations, orthopedic surgeons may hesitate to inject dexamethasone intravenously for diabetic patients due to safety concerns. Previously, scholars have studied the safety aspects of perioperative administration of glucocorticoids including dexamethasone and methylprednisolone in non-diabetic patients undergoing TJA. They concluded that glucocorticoids did not increase the rates of PJI and wound complications [31–34]. Moreover, other scholars examined the influence of dexamethasone on PJI and wound complications in diabetic patients undergoing TJA [18, 19]. Their findings indicated that both the incidences of PJI and wound complication were low, with no difference between the dexamethasone and non-dexamethasone groups within 90 days postoperatively. Our results showed that only 5 patients encountered wound complications and 2 patients encountered PJI within 90 days postoperatively, all of which were from non-dexamethasone group. And no differences were observed among the three cohorts. Our study has come to the same conclusion regarding the PJI and wound complications. Furthermore, in a larger cohort receiving dexamethasone, we firstly demonstrated there were no differences on the postoperative complications in preoperative administration of 5 mg or 10 mg dexamethasone.

The study has several limitations. First, this study relied on retrospective analysis of discharge records, potentially introducing biases associated with data quality. High-quality studies such as RCTs about the effect of dexamethasone on FBG levels in diabetic patients undergoing TJA should be conducted in the further to provide more reliable conclusions. Second, we just reported the clinical outcomes within 90 days postoperatively. And we did not acknowledge whether the rates of PJI and wound complications would undergo changes. Third, as our postoperative management has evolved, our hospital stay was short. This limited the data that were available for postoperative time points regarding blood glucose levels in our study. So, we can’t get the FBG changes in the subsequent time. We think with the establishment of national joint registration systems and further development of monitoring devices on blood glucose, these two limitations can be resolved in the further. Last, high-dose and repetitive dose administration of steroids in TJA patients was gradually common [35, 36], but our study was limited to administration of single and relatively low-dose dexamethasone. Moreover, if the study was conducted with other types and dose of steroids, the results might be different.

## Conclusions

Preoperative intravenous administration of 5 mg or 10 mg dexamethasone to diabetic patients has transient effects on increasing the blood glucose levels after TJA. Notably, patients with a preoperative HbA1c level of ≥ 7.15% and elevated preoperative FBG may be associated with postoperative glucose levels ≥ 200 mg/dl, regardless of dexamethasone administration and the dose. In this study population, preoperative intravenous administration of 5 mg or 10 mg dexamethasone to diabetic patients undergoing TJA may not increase the rates of PJI and wound complications within 90 days postoperatively.

## Data Availability

The analyzed dataset in this study is available from the first author on reasonable request.

## Abbreviations

DM: Diabetes mellitus
TJA: Total joint arthroplasty
THA: Total hip arthroplasty
TKA: Total knee arthroplasty
PJI: Prosthetic joint infection
HbA1c: Glycated hemoglobin
FBG: Fasting blood glucose
POD: Postoperative day
SD: Standard deviation
OR: Odds ratios
CI: Confidence intervals
AUC: Area under the curve
